# Interference with jasmonic acid-regulated gene expression is a general property of viral suppressors of RNA silencing but only partly explains virus-induced changes in plant–aphid interactions

**DOI:** 10.1099/vir.0.060624-0

**Published:** 2014-03

**Authors:** Jack H. Westwood, Mathew G. Lewsey, Alex M. Murphy, Trisna Tungadi, Anne Bates, Christopher A. Gilligan, John P. Carr

**Affiliations:** Department of Plant Sciences, Downing Street, University of Cambridge, Cambridge CB2 3EA, UK

## Abstract

The cucumber mosaic virus (CMV) 2b viral suppressor of RNA silencing (VSR) inhibits host responses to jasmonic acid (JA), a chemical signal regulating resistance to insects. Previous experiments with a CMV subgroup IA strain and its *2b* gene deletion mutant suggested that VSRs might neutralize aphid (*Myzus persicae*) resistance by inhibiting JA-regulated gene expression. To further investigate this, we examined JA-regulated gene expression and aphid performance in *Nicotiana benthamiana* infected with Potato virus X, Potato virus Y, Tobacco mosaic virus and a subgroup II CMV strain, as well as in transgenic plants expressing corresponding VSRs (p25, HC-Pro, 126 kDa and 2b). All the viruses or their VSRs inhibited JA-induced gene expression. However, this did not always correlate with enhanced aphid performance. Thus, VSRs are not the sole viral determinants of virus-induced changes in host–aphid interactions and interference with JA-regulated gene expression cannot completely explain enhanced aphid performance on virus-infected plants.

Most plant viruses encode at least one viral suppressor of RNA silencing (VSR) (Voinnet *et al.* 1999). Among the first VSRs discovered was the 2b VSR encoded by cucumber mosaic virus (CMV) (Brigneti *et al.*, 1998). The 2b VSR inhibits silencing predominantly through sequestration of small dsRNAs (Goto *et al.*, 2007; Chen *et al.*, 2008; González *et al.*, 2010, 2012). Additionally, the 2b VSR can directly interact with and inhibit the ARGONAUTE (AGO) proteins AGO1 and AGO4 (Zhang *et al.*, 2006; González *et al.*, 2010; Harvey *et al.*, 2011). In *Arabidopsis thaliana*, the 2b VSR encoded by a subgroup IA CMV strain (Fny-CMV) interacted strongly with AGO1 and interfered with microRNA-regulated gene expression and plant development, whereas 2b proteins of subgroup II strains (LS-CMV and Q-CMV) did not interact as strongly with AGO1 or induce such strong developmental defects in the host (Zhang *et al.*, 2006; Lewsey *et al.*, 2007). However, these differences must depend to a certain extent upon the host plant, as it was found that in tomato (*Solanum lycopersicum*) the LS-CMV 2b VSR inhibited microRNA-mediated regulation of gene expression (Cillo *et al.*, 2009).

The 2b VSR also interferes with signalling mediated by salicylic acid (Ji & Ding, 2001), abscisic acid (Westwood *et al.*, 2013a) and jasmonic acid (JA) (Lewsey *et al.*, 2010). Remarkably, transgenic expression of the 2b VSR in *A. thaliana* inhibited the normal responses of over 90 % of JA-regulated plant genes to methyljasmonic acid (MeJA) treatment (Lewsey *et al.*, 2010). Among other things, JA orchestrates responses to herbivores including aphids (Ellis *et al.*, 2002; Wasternack, 2007; Rohwer & Erwin, 2008; Bari & Jones, 2009). It was therefore suggested that the 2b protein inhibits resistance to these insects (Lewsey *et al.*, 2010), which are vectors for CMV and many other plant viruses (Palukaitis & García-Arenal, 2003; Westwood & Stevens, 2010). Subsequently, it was shown that in tobacco (*Nicotiana tabacum*) plants, the 2b VSR inhibits induction of resistance to the aphid *Myzus persicae* by other viral gene product(s) during infection by Fny-CMV (Ziebell *et al.*, 2011). In contrast, it was reported that VSRs encoded by certain other aphid-transmitted viruses have the ability to enhance JA-mediated signalling: HC-Pro derived from the potyvirus tabacco etch virus and the P6 protein from cauliflower mosaic virus enhanced responses to JA when expressed in transgenic *A. thaliana* (Endres *et al.*, 2010; Love *et al.*, 2012). However, for the whitefly-transmitted begomovirus tomato yellow leaf curl China virus, the inhibitory effect on JA-regulated gene expression of the βC1 pathogenicity factor encoded by its satellite DNA appears to explain increased whitefly performance on infected plants (Zhang *et al.*, 2012). JA-mediated signalling can also be disrupted by geminiviruses via disruption of jasmonate perception, as in the case of the C2 transactivator protein of tomato yellow leaf curl Sardinia virus (Lozano-Durán *et al.*, 2011).

The molecular mechanisms governing vector–plant interactions in virus-infected hosts are poorly understood, and it is likely that there are important host-specific effects at work. Thus, by contrast with its effects in tobacco (Ziebell *et al.*, 2011), Fny-CMV induced a mild resistance to feeding by *M. persicae* in squash (*Cucurbita pepo*) plants (Mauck *et al.*, 2010). As CMV is transmitted in a non-persistent manner, in which virus acquisition is favoured by short feeding periods and not by prolonged ingestion, this may aid transmission between squash plants (Mauck *et al.*, 2010). Fny-CMV also induced in squash the increased production of aphid-attracting volatiles (Mauck *et al.*, 2010), as did infection of potato (*Solanum tuberosum*) by potato leafroll virus, which is persistently transmitted by aphids (Eigenbrode *et al.*, 2002).

It was not entirely clear from these previous studies what roles specific viral gene products play in shaping host–aphid interactions. For example, is it always VSRs that shape changes in host interactions with insects? It was also not clear if only insect-transmitted viruses alter JA-responsive gene expression. Therefore, we compared the effects on JA-responsive gene expression and aphid performance of virus infection and transgenic VSR expression for aphid-transmitted viruses [CMV and the potyvirus potato virus Y (PVY)] and for two viruses that are mechanically transmitted and are not known to have any insect vectors [the tobamovirus tobacco mosaic virus (TMV) and the potexvirus potato virus X (PVX)]. An additional reason for choosing PVY was that previous work had indicated that this virus perturbs JA-mediated signalling (Kovač *et al.*, 2009).

*N. benthamiana* plants infected with LS-CMV (a subgroup II CMV strain), Fny-CMV, PVY, PVX and TMV were sprayed with 250 µM MeJA and tissue was harvested at 0, 6 and 24 h post-treatment (Fig. 1). MeJA-responsive transcripts were identified from a previously published microarray study, which assessed responses to MeJA in *A. thaliana* (Lewsey *et al.*, 2010). The TIGR plant transcript assemblies database (http://plantta.jcvi.org/) was used to identify *N. benthamiana* homologues for selected genes and these were confirmed to be JA-responsive (Fig. 1). Lipoxygenase 2 (LOX2) is an enzyme involved in an early step of JA biosynthesis and its own gene is regulated by JA as part of a positive feedback loop that perpetuates the defence signal (Wasternack, 2007). The transcripts *DEFENSIN 2.2* (*DEF2.2*) and *TRYPSIN PROTEIN INHIBITOR* (*TPI*) are key downstream outputs of the JA pathways, encoding proteins that function in anti-insect defence. Also monitored was an *N. benthamiana* orthologue of the JA-inducible *A. thaliana* transcript *AT3G55290*, which encodes a putative protein of no known function in the NAD(P)-binding Rossmann-fold superfamily (Lewsey *et al.*, 2010). PCR primer information is provided in Table S1 (available in the online Supplementary Material). Two housekeeping genes, *elongation factor 1α* (*EF1α*) and *glyceraldehyde-3-phosphate dehydrogenase*, were identified during preliminary work as unresponsive to MeJA treatment and suitable for use as internal PCR standards. Reactions were routinely normalized to expression of *EF1α* (Table S2 and Spreadsheet S1).

**Fig. 1.**
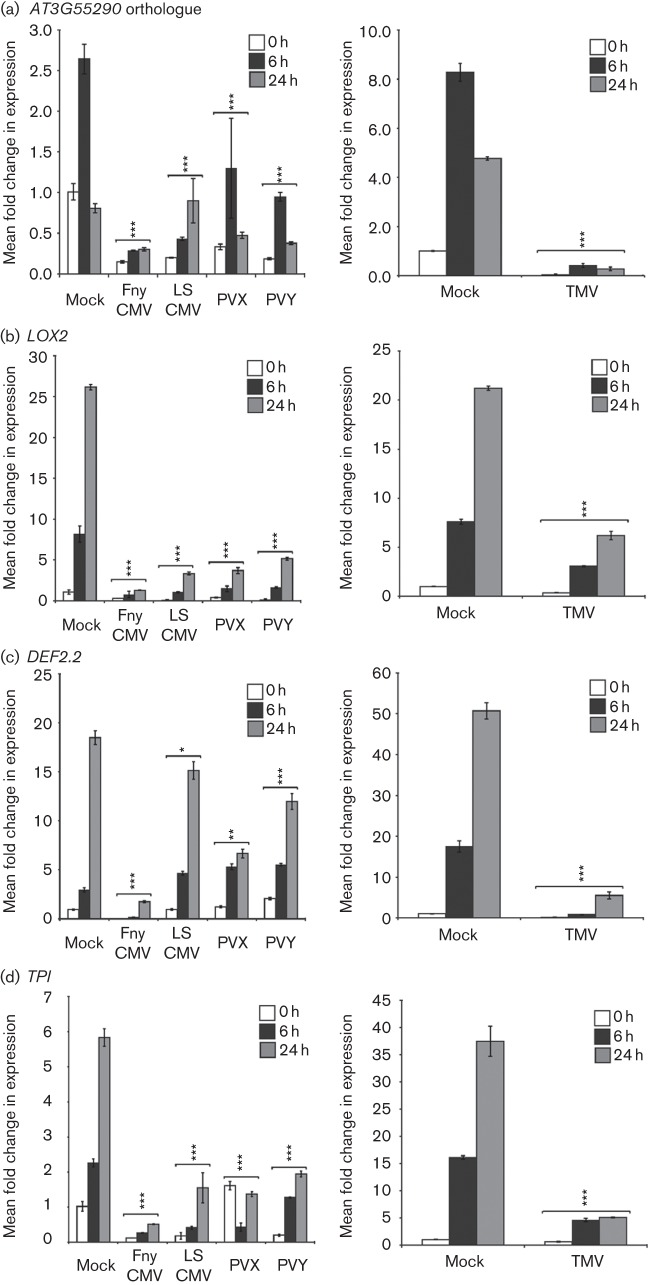
Infection by a range of viruses inhibited JA-mediated gene expression. *N. benthamiana* plants systemically infected with either Fny-CMV, LS-CMV, PVX, PVY or TMV were treated with MeJA at 2 weeks post-inoculation. Aerial tissue from three plants per treatment group was harvested immediately prior to treatment (0 h) and at 6 and 24 h following treatment. RNA was extracted from pooled tissue samples and reverse transcription coupled to quantitative PCR was performed to measure the transcript abundance of the JA-regulated transcripts *AT3G55290* (a), lipoxygenase 2 (*LOX2*) (b), *DEFENSIN 2.2* (*DEF2.2*) (c) and *TRYPSIN PROTEINASE INHIBITOR* (*TPI*) (d). Transcript accumulation levels were compared with those in mock-inoculated (Mock) plants. Significant suppression of MeJA-induced gene expression changes at one or both post-treatment time points are indicated (*t*-test: *P<0.05, **P<0.01, ***P<0.001). Histogram bars represent RNA samples from three plants (technical replicates). Error bars represent sem.

We used reverse transcription coupled to quantitative PCR to assess relative transcript abundance in infected plants following MeJA treatment. These and all other experiments in this study were performed at least three times independently. In all cases, virus infection inhibited induction of all JA-regulated genes tested. In most cases, the basal accumulation of JA-regulated transcripts was depressed in virus-infected plants (Fig. 1). There appeared to be no relationship between inhibition of JA-regulated gene expression by a virus and its transmissibility by aphids. For example, MeJA-induced expression of the *N. benthamiana AT3G55290* orthologue, *LOX2*, *DEF2.2* and *TPI* was inhibited by infection with TMV and PVX, neither of which is transmitted by aphids, as well as by Fny-CMV, LS-CMV and PVY, which are aphid-transmitted viruses (Fig. 1).

MeJA was applied to transgenic plants expressing VSRs in three plant lines described by Siddiqui *et al.* (2008): p25 (from PVX) (Chiu *et al.*, 2010), HC-Pro (from PVY) (Anandalakshmi *et al.*, 1998; Brigneti *et al.*, 1998) and 2b (from the subgroup II CMV strain KIN); or the 126 kDa replication protein/VSR (Kurihara *et al.*, 2007; Vogler *et al.*, 2007) encoded by TMV (described by Harries *et al.*, 2008). KIN-CMV is, like LS-CMV, classified as a subgroup II CMV strain (Palukaitis & García-Arenal, 2003) and the amino acid sequences of the 2b proteins of LS-CMV and KIN-CMV are identical. The responses of all four JA-regulated transcripts to MeJA treatment were markedly depressed in plants of lines expressing p25, HC-Pro, and 126 kDa proteins (Fig. 2). However, infection with the corresponding viruses inhibited expression to a greater degree (Fig. 1). It appeared that expression of the 2b protein derived from KIN-CMV did not significantly inhibit accumulation of JA-responsive transcripts following application of MeJA in contrast to the other VSRs tested (Fig. 2). The ability of the PVY-derived HC-Pro to inhibit JA-responsive gene expression in *N. benthamiana* contrasts with the reported effects of *Tobacco etch virus* in *A. thaliana* (Endres *et al.*, 2010).

**Fig. 2.**
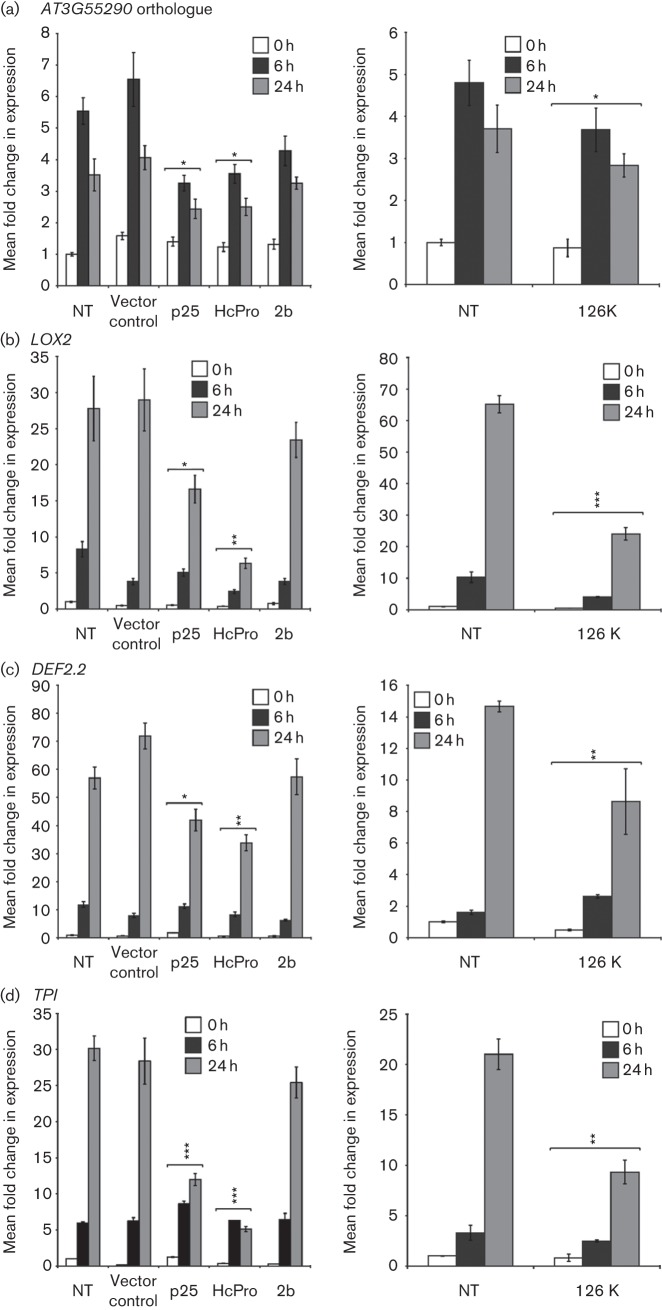
Transgenic expression of VSRs inhibits JA-induced gene expression in *N. benthamiana*. Non-transgenic (NT) and transgenic *N. benthamiana* plants expressing p25, HC-Pro or 2b or transformed with an ‘empty’ vector (vector control line) were treated with MeJA. Aerial tissue from three plants per treatment group was harvested immediately prior to treatment (0 h) and at 6 and 24 h following treatment. RNA was extracted from pooled tissue samples and reverse transcription quantitative PCR was performed to measure the transcript abundance of the JA-regulated transcripts for the *N. benthamiana* orthologue of *AT3G55290*, *LOX2*, *DEF2.2* and *TPI.* Significant suppression of MeJA-induced gene expression changes at one or both post-treatment time points is indicated (*t*-test: *P<0.05, **P<0.01, ***P<0.001). Histogram bars represent RNA samples from three plants (technical replicates). Error bars represent sem.

To test whether these viruses or the VSRs they encoded affected aphid performance on plants, we measured aphid growth rates and monitored aphid colony development on virus-infected plants or VSR-expressing transgenic plants (Fig. 3). A colony of *M. persicae* clone US1L (Devonshire & Sawicki, 1979) was established on *N. benthamiana*. To determine growth rates, 1-day-old nymphs were individually weighed on a microbalance (MX5; Mettler-Toledo), then placed on test plants and reweighed 5 days later. The mean relative growth rate (MRGR) was calculated as described previously (Leather & Dixon, 1984; Ziebell *et al.*, 2011). Aphid colony growth was measured by counting the progeny of a single nymph 12 days after its placement on a plant.

**Fig. 3.**
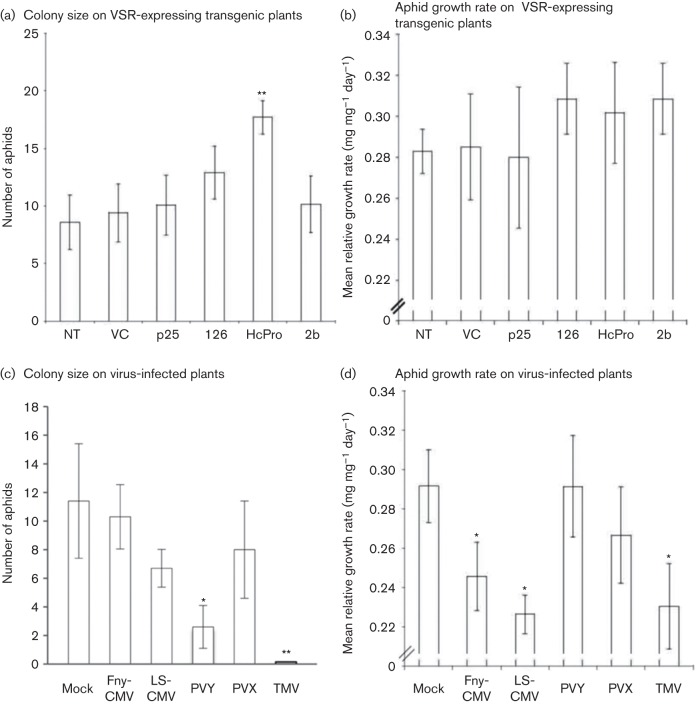
Effects of VSR expression versus virus infection on aphid performance. (a) Aphid colony development was enhanced on *N. benthamiana* plants expressing HC-Pro derived from PVY. A 1-day-old nymph was placed on a leaf in a clip cage and its progeny counted after 12 days. (b) Mean relative growth rate (MRGR) of aphids was unaltered on transgenic plants expressing VSR proteins derived from PVX (p25), TMV (126 kDa), PVY (HC-Pro) or CMV strain KIN (2b), or on plants transformed with an ‘empty’ vector control (VC) or non-transformed (NT) plants. Nymphs were weighed prior to being placed on the plant and after 5 days. MRGR was calculated according to the method of Leather & Dixon (1984). (c) Colony growth on virus-infected plants was determined as in (a). (d) MRGR for aphids on virus-infected plants. A 1-day-old nymph was weighed and placed contained in a clip cage on a leaf of an infected plant 5 days post-inoculation, reweighed 5 days later and MRGR calculated as in (b). Means of test groups (placed on infected or transgenic plants) and mean values for aphids placed on mock or non-transgenic plants were compared by *t*-tests: *P<0.05; **P<0.01. Error bars represent sem.

Aphid colony growth was not affected to any significant degree on *2b*-transgenic, *126 kDa*-transgenic or *p25*-transgenic plants but was significantly enhanced on plants expressing HC-Pro (Fig. 3a). We also measured MRGR for aphids placed on these transgenic plants but found no significant differences (Fig. 3b). The positive effect of HC-Pro expression on aphid colony growth was not reflected in colony growth data from PVY-infected plants; infection with PVY markedly inhibited aphid reproduction (Fig. 3c). Infection of host plants with Fny-CMV, LS-CMV and PVX did not significantly inhibit aphid colony growth (Fig. 3c), although the growth rates for aphids placed on plants infected with Fny-CMV and LS-CMV were depressed (Fig. 3d). Aphid growth rates were not inhibited on PVY-infected plants (Fig. 3d). Aphid growth and colony production were both markedly decreased on TMV-infected plants (Fig. 3c, d), but this was most likely due to the initiation of systemic necrosis, which is a feature of TMV infection in *N. benthamiana*.

We have shown that infection by TMV, PVX, PVY and CMV inhibits induction of JA-dependent genes in *N. benthamiana* plants. However, the effect was seen not only during infection by aphid-transmitted viruses (CMV and PVY), but also during infection by mechanically transmitted viruses (TMV and PVX). Therefore, there is no simple correlation between the ability of a virus or its corresponding VSR to interfere with JA-induced host gene expression with the mode of transmission of that virus. The aphid-transmitted viruses PVY and CMV did affect aphid performance, with effects on colony size and growth rate, respectively. This may indicate some similarities with the model proposed by Mauck *et al.* (2010) for CMV-infected squash in which host quality was diminished by the virus to favour migration of aphids, leading to increased CMV transmission. It is notable, however, that expression of HC-Pro in transgenic plants increased aphid colony growth, whereas PVY infection inhibited it. These conflicting observations lead to two conclusions. First, as transgenic HC-Pro expression and PVY infection both inhibited JA-regulated gene expression but only HC-Pro expression increased aphid performance, virus-induced changes in host–aphid interactions cannot be explained purely by inhibition of JA-mediated gene expression. Secondly, the results indicate, as do recent experiments with Fny-CMV in *A. thaliana* (Westwood *et al.*, 2013b), that virus-induced changes in host–aphid interactions are not solely regulated by VSRs but must be conditioned by the direct or indirect interactions of more than one viral gene product with the host and, perhaps, with each other.
